# Selective HDAC4 inhibition by SP1-PTD promotes odontoblast differentiation

**DOI:** 10.1590/1678-7757-2025-0447

**Published:** 2025-12-01

**Authors:** Yeo-Kyeong SHIN, Jung-Sun MOON, Su-Kyeong SON, Bin-Na LEE, Chungoo PARK, Sun-Hun KIM, Young Chul LEE, Min-Seok KIM

**Affiliations:** 1 Chonnam National University School of Dentistry Dental Science Research Institute Gwangju Republic of Korea Chonnam National University, School of Dentistry, Dental Science Research Institute, Gwangju, Republic of Korea.; 2 Chonnam National University School of Biological Sciences and Technology Gwangju Republic of Korea Chonnam National University, School of Biological Sciences and Technology, Gwangju, Republic of Korea.

**Keywords:** Runt-related transcription factor 2, Histone deacetylase inhibitors, Odontoblast, Dentin sialophosphoprotein, Peptides

## Abstract

**Background:**

Vital pulp therapy is limited by incomplete dentin regeneration and dose-limiting toxicities of current histone deacetylase (HDAC) inhibitors. Previous structural studies have identified critical determinants of HDAC4-silencing mediator for retinoid and thyroid hormone receptor (SMRT) protein interactions, providing a rationale for developing selective inhibition strategies.

**Objective:**

This study evaluated SMRT peptide 1-protein transduction domain (SP1-PTD), which is a cell-penetrating peptide designed to selectively disrupt HDAC4–SMRT interaction based on structural insights, for promoting odontoblast differentiation with improved safety compared to pan-HDAC inhibitors.

**Methodology:**

SP1-PTD comprises an SMRT-derived sequence fused to a PTD, enabling targeted inhibition without affecting HDAC catalytic activity. Effects on odontoblast differentiation were assessed in murine dental papilla cell lines and primary human dental pulp cells using gene expression analysis, functional mineralization assays, and mechanistic studies including chromatin immunoprecipitation and RUNX2 acetylation analysis. Cytotoxicity was directly compared with suberoylanilide hydroxamic acid (SAHA) and trichostatin A.

**Results:**

SP1-PTD treatment significantly enhanced odontoblast differentiation with 15.9-fold increase in dentin sialophosphoprotein (Dspp) expression alongside upregulation of RUNX2, osteocalcin, and bone sialoprotein. Functional analysis revealed 1.8-fold increased mineralization capacity. Mechanistically, SP1-PTD increased RUNX2 protein acetylation and histone acetylation at the Dspp promoter, indicating derepression of RUNX2-mediated transcription. Importantly, SP1-PTD did not show cytotoxicity across a wide therapeutic range (0.1-20 μM) and promoted cell proliferation, contrasting sharply with dose-dependent toxicity of pan-HDAC inhibitors. Direct comparison revealed SP1-PTD induced 14-fold increase in Dspp expression while SAHA suppressed it despite comparable *Runx2* induction.

**Conclusions:**

SP1-PTD represents a first-in-class selective HDAC4 inhibitor that achieves robust pro-differentiation effects with an exceptional safety profile. By specifically targeting HDAC4–SMRT interactions, SP1-PTD overcomes limitations of conventional HDAC inhibitors and offers translational promise for dental regenerative medicine.

## Introduction

Regeneration of the dentin-pulp complex remains a critical unmet need in regenerative dentistry. Current therapeutic agents, such as calcium hydroxide and mineral trioxide aggregate, have limited regenerative capacity and frequently result in incomplete dentin bridge formation. Moreover, these materials often fail to achieve true pulp regeneration, instead forming calcified barriers that may compromise long-term pulp vitality and function.^1^ Thus, developing bioactive agents that can promote complete odontoblast differentiation and functional dentin regeneration while maintaining a favorable safety profile remains a major challenge.

Odontoblast differentiation is primarily regulated by Runt-related transcription factor 2 (RUNX2)/core-binding factor alpha 1, which is a master regulator originally identified in osteoblast-mediated bone formation.^2^ RUNX2 governs odontoblast differentiation in dental tissues by regulating key odontogenic genes, particularly dentin sialophosphoprotein (*Dspp*).^3^ *Dspp*, which is predominantly expressed in mature odontoblasts, encodes dentin sialoprotein (DSP), a critical non-collagenous protein that functions as an odontoblast-specific marker and a nucleator of dentin mineralization.^4^ RUNX2 further supports this differentiation program by inducing the expression of other mineralization-related genes, including dentin matrix protein 1 (*Dmp 1*), bone sialoprotein (*Bsp*), and osteocalcin (*Ocn*).^5,6^

The transcriptional activity of RUNX2 is tightly regulated by post-translational modifications, particularly acetylation, which enhances RUNX2 protein stability and transcriptional function, whereas deacetylation facilitates its degradation.^7^ This regulation is mediated by histone deacetylases (HDACs), a family of 18 enzymes that remove acetyl groups from histones and non-histone proteins.^8^ Among these, HDAC4 plays an important role in osteoblast differentiation via mechanisms distinct from other HDAC family members.^9^ HDAC4, a class IIa HDAC, differs from class I HDACs because it lacks intrinsic catalytic activity. Instead, HDAC4 functions as a scaffold protein: its N-terminal adaptor domain binds transcription factors such as RUNX2, while its C-terminal domain recruits the HDAC3–silencing mediator for retinoid and thyroid hormone receptor (SMRT) complex.^10^ This interaction enables HDAC4 to mediate RUNX2 deacetylation indirectly via HDAC3, resulting in RUNX2 destabilization and transcriptional repression.^11^ Based on these mechanisms, HDAC4 likely serves as a critical negative regulator of odontoblast differentiation by suppressing RUNX2 activity.

This understanding has sparked significant interest in HDAC inhibitors as potential therapeutic agents for dental regeneration. Pan-HDAC inhibitors such as trichostatin A (TSA), valproic acid, sodium butyrate, and suberoylanilide hydroxamic acid (SAHA) have demonstrated the ability to enhance odontoblast differentiation *in vitro*.^12-15^ However, these compounds target the conserved catalytic domain shared by multiple HDAC isoforms,^16^ resulting in dose-limiting toxicities, including thrombocytopenia, cardiotoxicity, and anemia that prevented their clinical translation.^17-19^ The clinical application of these agents in vital pulp therapy is further complicated by the need for localized delivery to avoid systemic side effects, highlighting the urgent need for safer and more selective approaches.

Structural studies by Park, et al.^20^ (2018) provided critical insights into HDAC4 regulation by identifying the molecular determinants of HDAC4-SMRT interaction. These investigations revealed that SMRT peptide 1 (SP1), derived from the glycine-serine-isoleucine motif of SMRT-repression domain 3, specifically mediates binding to class IIa HDACs. Unlike catalytic inhibitors, SP1 selectively disrupts the HDAC4–SMRT protein–protein interaction without inhibiting HDAC enzymatic activity, representing a novel strategy for HDAC4 inhibition. Thus, we hypothesized that SP1-based inhibitors could be translated to dental regenerative applications, in which selective HDAC4 inhibition might promote odontoblast differentiation while avoiding the toxicities associated with pan-HDAC inhibition.

The potential clinical advantages of this selective approach are substantial. First, by preserving the catalytic functions of other HDAC isoforms, SP1-based inhibitors would avoid the systemic toxicities that have limited pan-HDAC inhibitor translation. Second, the peptide-based nature of SP1 derivatives offers advantages for localized delivery in dental applications, enabling direct application to pulp tissues via pulp capping materials or injectable hydrogels. Third, selective HDAC4 inhibition specifically targets the pathway most relevant to odontoblast differentiation, potentially achieving superior therapeutic efficacy with reduced off-target effects.

In this study, the SP1-protein transduction domain (PTD) was engineered by conjugating the SMRT-derived inhibitor peptide (SP1: IRGSITQGIPR) with a PTD sequence (PTD: YARVRRRGPRR) to enhance cellular uptake and to evaluate its potential as a safe and effective agent for dental pulp regeneration. The hypothesis was that SP1-PTD specifically alleviates HDAC4-mediated repression of RUNX2 by preventing the recruitment of the SMRT–HDAC3 complex, thereby promoting odontoblast differentiation with reduced toxicity compared to pan-HDAC inhibitors. This study aimed to assess the effects of SP1-PTD on odontoblast differentiation in murine and human dental pulp cells (HDPCs), to elucidate the underlying molecular mechanisms with particular emphasis on RUNX2 acetylation and target gene regulation, and to compare the efficacy and safety profiles of SP1-PTD with those of conventional HDAC inhibitors. Collectively, this research aims to establish a novel paradigm for dentin regeneration via the selective disruption of HDAC4 protein–protein interactions, while highlighting SP1-PTD as a clinically relevant therapeutic strategy that may overcome the safety limitations impeding the translation of HDAC-based therapies in dentistry.

## Methodology

### Peptide design and synthesis

The SP1-PTD peptide was designed based on structural insights from our previous crystallographic study of HDAC4-SMRT interaction.^20^ The SP1 sequence (IRGSITQGIPR) was derived from amino acids 1361-1369 of human SMRT-repression domain 3, corresponding to the critical glycine-serine-isoleucine motif that specifically mediates binding to class IIa HDACs. This sequence was selected based on the following criteria: (1) direct contact residues identified in the HDAC4-SMRT crystal structure, (2) conservation across species, and (3) demonstrated binding specificity for class IIa versus class HDACs in previous biochemical assays. The SP1 was fused at its N-terminus to a PTD sequence YARVRRRGPRR, derived from the human hypoxia-inducible protein 1 (Hph-1) to enable cellular permeability.^18^ This particular PTD was selected based on its established efficacy in peptide delivery applications and its minimal cytotoxicity profile compared to other cell-penetrating peptides such as TAT or polyarginine sequence.

The SP1-PTD fusion peptide (final sequence: YARVRRRGPRR- IRGSITQGIPR) was chemically synthesized using solid-phase peptide synthesis with Fmoc chemistry by Anygene Co. (Gwangju, South Korea). Peptide purity was confirmed to be >95% by high-performance liquid chromatography (HPLC), and identity was verified by mass spectrometry. The lyophilized peptide was reconstituted in dimethyl sulfoxide (DMSO) to prepare a 20 mM stock solution, which was aliquoted and stored at -80°C. Working concentrations were prepared by diluting the stock solution in medium culture to achieve final DMSO concentrations ≤0.1% to avoid solvent-related cytotoxicity. Peptide stability was confirmed for up to six months under these storage conditions by analytical methods. For quality control, each peptide batch was tested for: (1) purity by analytical HPLC, (2) identity by mass spectrometry, (3) endotoxin levels using standard assays, and (4) sterility by microbiological testing. Only peptide batches meeting all quality criteria were used in experiments.

### Murine dental papilla-derived cell line culture

Two cell lines derived from murine dental papilla, namely MDPC23 and mDP cells, were employed in this study. Both cell lines were cultured in Dulbecco’s modified Eagle medium (DMEM; 11995-065, Gibco BRL, Gaithersburg, MD, USA) supplemented with 10% fetal bovine serum (FBS; 16000-044, Gibco BRL), 100 U/mL penicillin, and 100 μg/mL streptomycin, and maintained at 37℃ in a humidified incubator with 5% carbon dioxide (CO_2_). To induce odontoblastic differentiation, MDPC23 cells were cultured in a differentiation medium comprising DMEM supplemented with 5% FBS, 10 mM β-glycerophosphate (G9422, Sigma-Aldrich), and 50 μg/mL ascorbic acid 2-phosphate (A8960, Sigma-Aldrich), with or without SP1-PTD for the indicated durations. The medium was further supplemented with 200 ng/mL recombinant human bone morphogenic protein 2 (355-BM, R&D systems) for odontogenic differentiation of mDP cells. Based on the results of a pilot study (Supplement Figure 1), SP1-PTD was used at concentrations of 10 or 20 µM in subsequent experiments, with 20 µM being employed as the primary condition.

**Figure 1:**
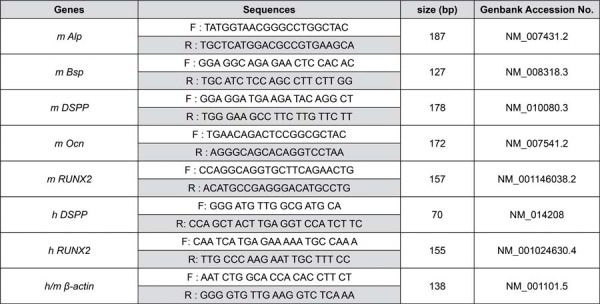
Oligonucleotide sequences of primers

(*m*, mouse; *h*, human)

### Primary HDPC culture

To ensure translational relevance and to confirm that the effects of SP1-PTD were not limited to murine-derived cells, primary human dental pulp cells (HDPCs) were employed in addition to murine cell models. HDPCs were isolated from healthy donors aged 16–25 years, following informed consent. The study protocol was approved by the Institutional Review Board (IRB) of Chonnam National University Medical School (IRB number: CNUDH-2016-009). Dental pulp tissues were aseptically harvested from freshly extracted teeth from five donors, pooled, minced, and incubated with 30 mg/mL collagenase type I (C0130, Sigma-Aldrich Co.) for 1 h at 37℃. The dissociated cells were cultured in α-modified Eagle’s medium (12561, GIBCO BRL) supplemented with 10% FBS, 100 U/mL penicillin, and 100 μg/mL streptomycin, and maintained in a humidified atmosphere of 5% CO_2_ at 37℃. HDPCs at passages two to three were used for experiments. Characterization of the cultured cells confirmed that they retained a mesenchymal and undifferentiated phenotype (Supplement Figure 2). Moreover, all cultures tested negative for mycoplasma contamination, ensuring the quality and reproducibility of the cell model (Supplement Figure 3). Odontoblastic differentiation was induced using a medium containing 10 mM β-glycerophosphate, 50 μg/mL ascorbic acid 2-phosphate, and 0.1 μM dexamethasone (D1756, Sigma-Aldrich Co.), with or without SP1-PTD.

**Figure 2:**
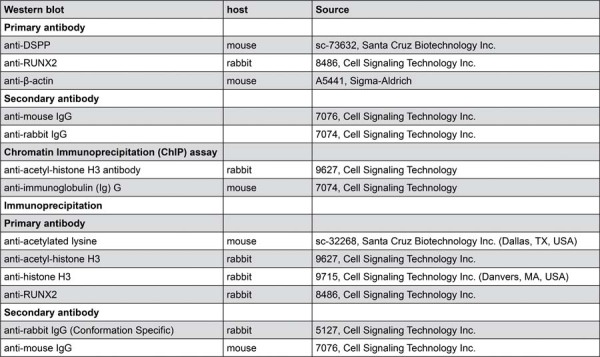
Antibodies used in this study

**Figure 3:**
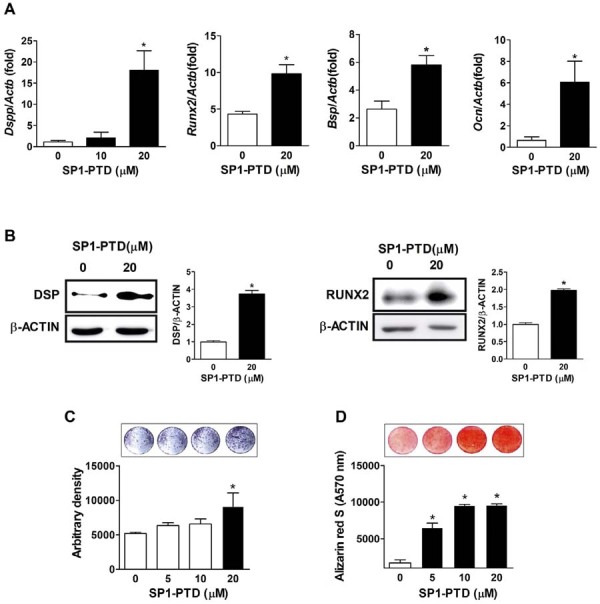
Effects of silencing mediator for the retinoid and thyroid receptor peptide 1 fused with protein transduction domain (SP1-PTD) on odontoblastic differentiation of MDPC23 cells. (A) MDPC23 cells were cultured in a differentiation medium with or without 20 μM SP1-PTD for five days. mRNA levels of dentin sialophosphoprotein (Dspp), Runt-related transcription factor 2 (*Runx2*), bone sialoprotein (Bsp) and osteocalcin (Ocn) were measured by real-time PCR. (B) Protein expression of dentin sialoprotein (DSP) and RUNX2 was analyzed by Western blotting following treatment with 20 μM SP1-PTD for five days. Cells were treated with 0, 5, 10, or 20 μM SP1-PTD in mineralization medium, followed by alkaline phosphatase staining after seven days (C) and Alizarin Red S staining after 12 days (D). Data are shown as mean ± standard deviation. * indicates statistically significant differences from the control (Mann–Whitney U test, p<0.05, n=3).

### Cytotoxicity assay

Cell viability was evaluated using the EZ-Cytox assay kit (Dogenbio Co., Ltd., Seoul, Korea). MDPC23 cells (8×10^3^ cells/well) were seeded in 48-well plates and incubated for 24 h, and were then treated with varying concentrations of the test reagents (0-1.0 µM SAHA, 0-20 μM TSA, 0-50 µM SP1-PTD) for an additional 24 h. The working concentrations of TSA and SAHA were selected based on established literature.^13,14^Then, EZ-Cytox reagent was added to each well, and the plates were incubated at 37°C for 30 min. Absorbance was subsequently measured at 450 nm. All experiments were performed with at least three independent biological replicates, each in technical triplicate.

### RNA preparation and real-time quantitative polymerase chain reaction (RT-qPCR)

Cells were seeded in 6-well plates at a density of 1-2×10^5^ cells per well and treated with 20 μM SP1-PTD. The treatment duration was five days for murine cells and seven to ten days for HDPCs. Total RNA was extracted using Trizol reagent (Thermo Fisher Scientific) according to the manufacturer’s instructions. Reverse transcription was performed using Moloney murine leukemia virus reverse transcriptase (M1705, Promega). Real-time complementary DNA amplification was performed using SYBR Green PCR Master Mix (QS105, Philekorea, Seoul, Korea) on a Rotor-Gene 3000 System (Qiagen). The PCR protocol comprised incubation at 95°C for 2 min, followed by 45 cycles of denaturation at 95°C for 5 s and annealing/extension at 62°C for 30 s. Figura 1 lists the primer sequences used in the analysis.

### Western blotting assay

Cells were seeded in 6 cm dishes at a density of 2–3 × 10^5^ cells per well and treated with 20 μM SP1-PTD for five days in murine cells and for ten days in HDPCs. Total protein was extracted using CytoBuster Protein Extraction Reagent (71009, Novagen), and were separated by 10-12% SDS-polyacrylamide gel electrophoresis and transferred onto nitrocellulose membranes using iBlot2 (Thermo Fisher Scientific., Waltham, MA, USA) for both total lysates and immunoprecipitated samples. Membranes were blocked with 5% skim milk in 10 mM Tris-buffered saline containing 0.1% Tween-20 for 1 h at room temperature, then incubated overnight at 4°C with primary antibodies. After washing, the membranes were incubated with horseradish peroxidase (HRP)-conjugated secondary antibodies for 1 h at room temperature. Subsequently, the blots were washed and developed using an HRP substrate (Millipore Corp., Billerica, MA, USA). Protein bands were visualized using the ODYSSEY imaging system (LI-COR, Lincoln, NE, USA) and quantified using Scion Image software. Figura 2 details the antibodies employed in the assay.

### Evaluation of odontoblast differentiation with alkaline phosphatase (ALP) staining and Alizarin red S staining

After treatment with 0–20 μM SP1-PTD during odontoblastic differentiation, ALP staining was performed on day seven for murine cell lines and day ten for primary HDPCs. For ALP staining, cells were washed with phosphate-buffered saline (PBS), fixed in 10% formaldehyde for 5 min, and rinsed with distilled water (DW). A 5-bromo-4-chloro-3-indolyl phosphate/nitro-blue tetrazolium solution (B1911, Sigma-Aldrich) was then added, and the enzymatic reaction was terminated by adding DW. Stained cells were imaged, and staining intensity was quantified using Scion Image software. Alizarin Red S staining was performed at time points corresponding to the stages of differentiation: at day 12 in murine cells and at day 21 in HDPCs. Cells were rinsed with PBS, fixed in 70% ethanol for 30 min at room temperature, and stained with 40 mM Alizarin red S (pH 4.2) for 10 min at room temperature. Images were captured after washing twice with DW, and the bound dye was extracted using 10% cetylpyridinium chloride (C5460, Sigma-Aldrich) for 15 min. Absorbance was measured at 570 nm using a microplate reader (Bio-Tek Instruments, Winooski, VT, USA).

### Luciferase reporter assay

*Dspp* promoter activity was evaluated using a luciferase reporter assay kit (Promega). MDPC23 cells were seeded in 24-well plates 24 h before transfection with Lipofectamine LTX and Plus Reagent (15338, Thermo Fisher Scientific), then were transfected with pGL3-*Dspp* with or without pFLAG-CMV5-RUNX2. After 24 h, cells were treated with 20 μM SP1-PTD for an additional 24 h, then, they were harvested, and luciferase activity was measured using a Lumat LB9501 luminometer (EG&G Berthold, BadWildbad, Germany), and normalized to β-galactosidase values.

### Chromatin IP (ChIP) assay

ChIP assay was performed using a ChIP Assay Kit (17-295, Millipore). Brieﬂy, MDPC23 cells (8×10^5^) were seeded in 10 cm culture dishes and cultured in differentiation medium with or without 20 μM SP1-PTD for 24 h. Cells were ﬁxed with 1% formaldehyde at 37°C for 10 min. Nuclei were isolated using a nuclear lysis buffer containing a protease inhibitor cocktail (Millipore). Chromatin deoxyribonucleic acid (DNA) was fragmented by sonication to 200–1000 base pairs. The sheared chromatin was diluted 1:10 in ChIP dilution buﬀer and incubated overnight at 4°C with anti-acetyl-histone H3 antibody or anti-immunoglobulin (Ig) G (Figura 2). Input DNA and non-speciﬁc IgG controls were included in each experiment. Protein A/G bead-antibody/chromatin complexes were washed and subjected to reverse cross-linking. The DNA was then purified and analyzed by polymerase chain reaction (PCR) using specific primers (forward: TTTCCCCGAGTCTGCATGAA; reverse: CTGAATTGTAAGCCAGCCTCA). PCR products were resolved by 1.5% agarose gel electrophoresis and visualized accordingly.

### Immunoprecipitation (IP) assay and immunoblotting

MDPC23 cells (3×10⁵) were seeded in 6-cm culture dishes and cultured in differentiation medium with or without 20 μM SP1-PTD for two days. Total proteins were extracted, and 80 μg of cell lysates were precleared with protein A/G agarose beads (GE Healthcare) for 30 min, followed by overnight incubation with the indicated primary antibodies. Fresh protein A/G agarose beads were subsequently added and incubated for 3 h at 4°C. After washing with extraction buffer, the beads were resuspended in 20 μL of SDS sample buffer and denatured at 95°C for 5 min. Eluted proteins were separated by 10–12% SDS–PAGE and transferred onto nitrocellulose membranes using the iBlot2 system (Thermo Fisher Scientific). For the detection of acetylated RUNX2, immunoprecipitation was performed using an anti–acetyl-lysine antibody, and the precipitated proteins were analyzed by immunoblotting with an anti-RUNX2 antibody. Immunoprecipitation was conducted using an anti–acetyl-histone H3 antibody to examine histone H3 acetylation, followed by immunoblotting with an anti-histone H3 antibody.

### Statistical analysis

All experiments were conducted with at least three independent biological replicates, each assessed in technical triplicates. Data normality could not be reliably evaluated due to the limited sample size (n=3 per group); Therefore, non-parametric statistical tests were employed. Data are shown as mean ± standard deviation (SD). Mann–Whitney U test was used for comparisons between two groups, and the Kruskal–Wallis test followed by Dunn’s post hoc test was used for comparisons between more than two groups. Statistical analyses were performed using GraphPad Prism (version 5.0; GraphPad Software, San Diego, CA, USA). Exact p-values are provided in the figure captions, with p<0.05 considered statistically significant.

## Results

### SP1-PTD promotes odontoblast differentiation of MDPC23 cell lines

The effects of SP1-PTD on odontoblast differentiation were examined using MDPC23 cells (Figure 3), which were cultured in a differentiation medium for five days with or without 20 μM SP1-PTD. The expression levels of odontoblast marker genes (*Dspp*, *Ocn*, *Bsp*, and *Runx2*) were assessed at the mRNA and protein levels. SP1-PTD treatment upregulated mRNA expression: *Dspp* (15.9-fold, p=0.0228), *Runx2* (2.2-fold, p=0.05), *Bsp* (2.1-fold, p=0.0383), and *Ocn* (9.3 fold, p=0.05), compared to controls (Figure 1A). Consistently, SP1-PTD also elevated expression protein levels of DSP and RUNX2 by 3.7 (p=0.05) and 2-fold (p=0.05), respectively (Figure 3B). To assess the mineralization-promoting effect of SP1-PTD, ALP activity was measured in MDPC23 cells cultured under differentiation conditions with or without various concentrations of SP1-PTD. Figure 3C shows that treatment with 20 μM SP1-PTD resulted in a 1.5-fold (p=0.05) increase in ALP activity. Alizarin red S staining further demonstrated a dose-dependent increase in mineralized nodule formation, with a 1.8-fold (p=0.05) increase at 20 μM SP1-PTD (Figure 3D).

### SP1-PTD also enhances odontoblast differentiation of mDP cell lines and primary HDPCs

**Figure 4:**
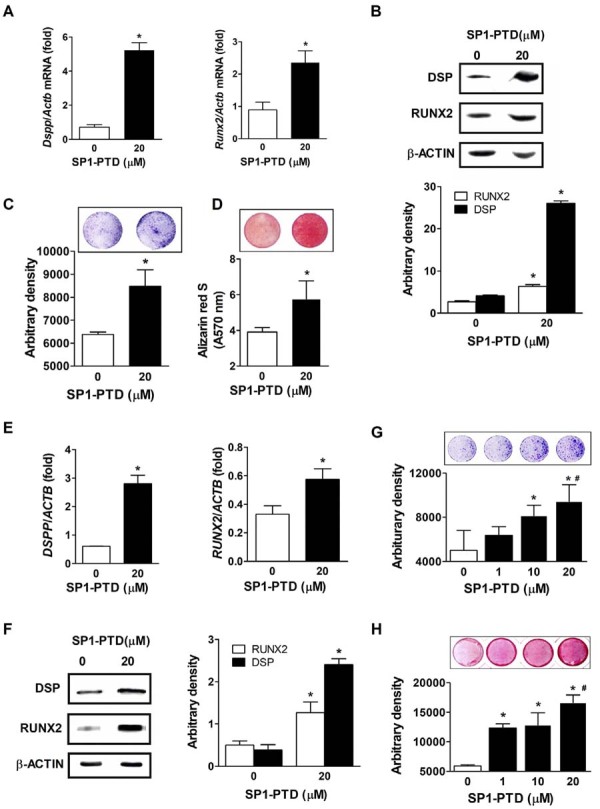
Effects of silencing mediator for the retinoid and thyroid receptor peptide 1 fused with protein transduction domain (SP1-PTD) on odontoblast differentiation of mDP cells and human dental pulp cells (HDPCs). (A) mDP cells were cultured in a differentiation medium with or without 20 μM SP1-PTD for five days. mRNA levels of dentin sialophosphoprotein (Dspp) and Runt-related transcription factor 2 (*Runx2*) were quantified by real-time PCR. (B) Under the same conditions, protein levels of dentin sialoprotein (DSP) and RUNX2 were analyzed by Western blotting in mDP cells. Mineralization was evaluated using alkaline phosphatase (ALP) staining on day seven (C) and Alizarin red S staining on day 12 (D) following SP1-PTD treatment. (E) HDPCs were cultured in odontoblast differentiation medium containing 0.1 μM dexamethasone with or without 20 μM SP1-PTD. The mRNA expression of RUNX2 and DSPP was measured on days seven and ten, respectively. (F) Protein levels of DSP and RUNX2 were assessed by Western blotting on day ten in HDPCs. (G-H) ALP staining (G) and Alizarin red S staining (H) were performed on days ten and 21, respectively in HDPCs. Data are shown as mean ± standard deviation (n=3). *p<0.05 by Mann–Whitney U test, #p<0.05 by Kruskal–Wallis test.

To validate the effect of SP1-PTD on odontoblast differentiation, its influence on the upregulation of odontoblast marker genes was further examined in mDP cells, another murine dental papilla-derived cell line (Figure 4). Following five days of odontogenic differentiation with or without 20 μM SP1-PTD treatment, mRNA levels of *Dspp* and *Runx2* in mDP cells were quantified (Figure 4A). SP1-PTD treatment resulted in 7.3-(p=0.0383) and 2.6-fold (p=0.05) increases in *Dspp* and *Runx2* mRNA levels, respectively, compared to untreated controls. Under the same conditions, DSP protein expression increased 6.3-fold (p=0.05), while RUNX2 protein levels showed a marginal increase (p=0.05) (Figure 4B). Enhanced mineralization in response to SP1-PTD was confirmed by ALP (p=0.0383) and Alizarin red S staining (p=0.05) (Figures 4C and 4D).

Subsequently, the effect of SP1-PTD on the induced differentiation of HDPCs was also evaluated to ensure translational relevance. On day seven post-induction, SP1-PTD treatment resulted in a 2.1-fold increase in *Runx2* mRNA levels, whereas on day ten, *Dspp* mRNA levels showed a 4.3-fold increase (Figure 4E). DSP and RUNX2 protein levels also increased significantly, showing an approximately 6.2 fold (p=0.05) and 2.5-fold (p=0.05) elevation under the same conditions (Figure 4F). SP1-PTD markedly enhanced mineralization during odontoblast differentiation of HDPC (Figures 4G and 4H).

### SP1-PTD stimulates RUNX2-mediated activation of *Dspp* promoter and RUNX2 acetylation

**Figure 5:**
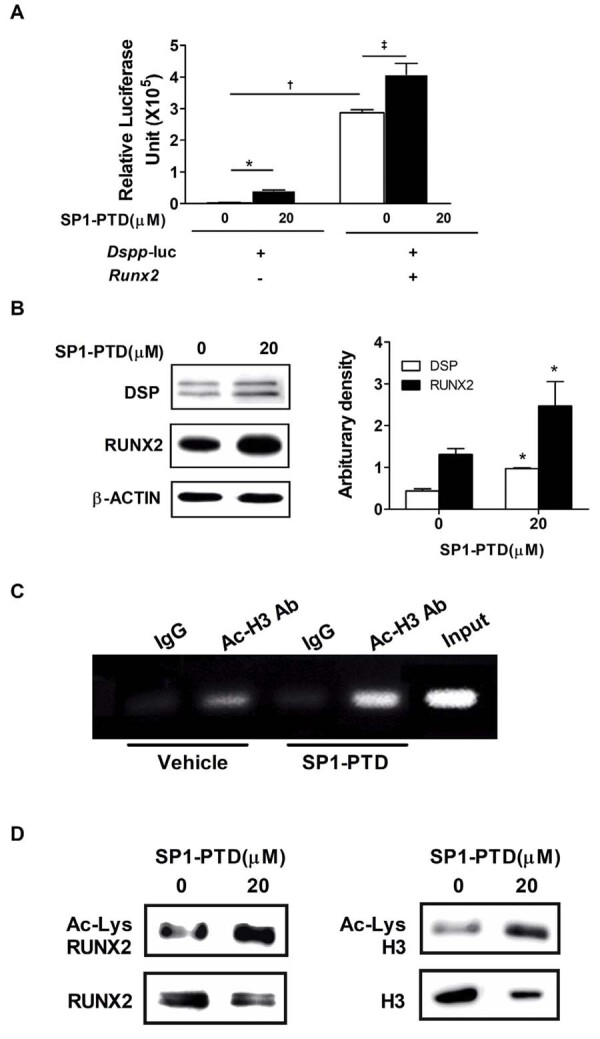
Silencing mediator for the retinoid and thyroid receptor peptide 1 fused with protein transduction domain (SP1-PTD) enhances Runt-related transcription factor 2 (RUNX2) transcriptional activity and acetylation, resulting in increased histone H3 acetylation at the dentin sialophosphoprotein (Dspp) promoter region. (A) The effect of SP1-PTD on Dspp promoter activity was assessed by luciferase reporter assay. MDPC23 cells were transfected with a Dspp-luciferase reporter plasmid with or without a *Runx2* expression vector, and treated with 20 μM SP1-PTD for 24 h. Promoter activity was quantified by luciferase assay. (B) Under the overexpression of RUNX2 protein, levels of RUNX2 and dentin sialoprotein (DSP) were assessed by Western blotting in MDPC23 cells. (C) The effect of SP1-PTD on histone H3 acetylation at the Dspp promoter region was analyzed by chromatin immunoprecipitation assay. MDPC23 cells were cultured in a differentiation medium with or without 20 μM SP1-PTD for 24 h. Chromatin DNA was immunoprecipitated using an antibody (IgG) or an antibody against acetylated histone H3. DNA was purified and subjected to PCR to monitor the presence of the Dspp promoter region (-2600 to -2361). (D) Acetylation of RUNX2 and histone H3 in MDPC23 cells was examined by immunoprecipitation after two days of differentiation induction with or without 20 μM SP1-PTD. Data are shown as mean ± standard deviation. * indicates statistically significant differences from the control (Mann–Whitney U test, p<0.05, n=3).

The hypothesis was that SP1-PTD upregulates odontoblast marker gene expression by relieving repression of RUNX2 transcriptional activity, given its function as an HDAC4-specific inhibitor. Thus, a luciferase reporter assay was performed using a *Dspp* promoter containing a RUNX2-binding site. MDPC23 cells were transiently transfected with the reporter plasmid, with or without *Runx2*, and luciferase activity was measured. As expected, *Runx2* expression increased *Dspp* promoter-driven luciferase activity by over 100-fold (p=0.05) (Figure 5A). Notably, 20 μM SP1-PTD further enhanced *Dspp* promoter activity in the presence or absence of *Runx2* (Figure 5A). In the absence of *Runx2* expression, SP1-PTD induced a 16-fold (p=0.05) increase in *Dspp* promoter activity, likely via activation of endogenous RUNX2. When *Runx2* was overexpressed, SP1-PTD led to a modest 1.4-fold (p=0.05) increase, possibly due to promoter saturation, which might have masked the additive effect of SP1-PTD. Under the overexpression of RUNX2 protein, SP1-PTD also elevated DSP and RUNX2 protein levels (Figure 5B). To further explore the mechanism of gene activation, ChIP assays were conducted to assess histone acetylation at the *Dspp* promoter. PCR targeting the *Dspp* promoter region (-2600 to -2361) showed a 3-fold increase in H3 acetylation at this locus (Figure 5C). These findings indicate that SP1-PTD enhances histone acetylation at the *Dspp* promoter, likely by preventing recruitment of the HDAC3–SMRT complex to RUNX2, thereby promoting transcription of odontogenic genes such as *Dspp* and *Runx2*.

RUNX2 protein stability is regulated by its acetylation status, therefore, the observed increase in RUNX2 protein levels following SP1-PTD treatment might result from enhanced acetylation. MDPC23 cells were treated with 20 μM of SP1-PTD and RUNX2 acetylation levels were evaluated to investigate this. Figure 5D shows that SP1-PTD treatment led to a 2-fold increase in RUNX2 acetylation, indicating inhibition of HDAC4-mediated deacetylation of RUNX2. These findings suggest that SP1-PTD upregulates RUNX2 protein levels via transcriptional activation of the *Runx2* gene and post-translational stabilization via increased acetylation, both likely mediated by HDAC4 inhibition via the SP1 peptide. Additionally, SP1-PTD induced a 1.6-fold increase in global histone H3 acetylation, further supporting its broader role in gene regulation by suppressing HDAC4-dependent transcriptional repression (Figure 5D).

### SP1-PTD promotes odontoblastic differentiation more efficiently with lower cytotoxicity than other pan-HDAC inhibitors

**Figure 6:**
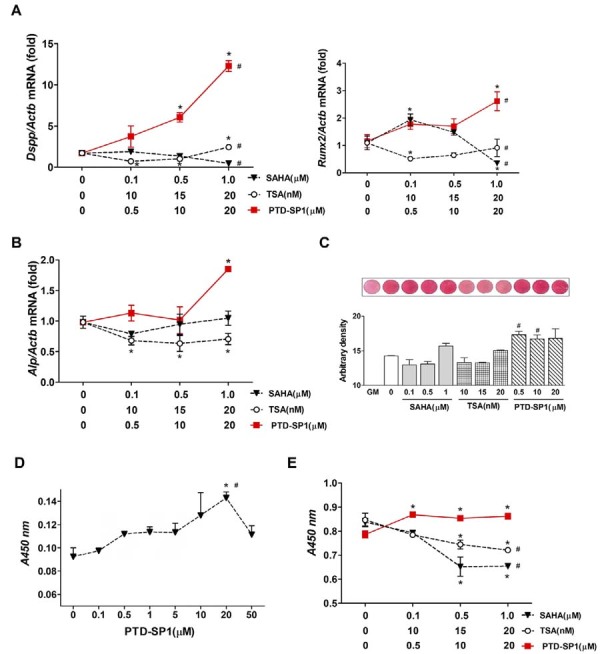
Silencing mediator for the retinoid and thyroid receptor peptide 1 fused with protein transduction domain (SP1-PTD) enhances odontoblast differentiation with reduced cytotoxicity compared to other pan-histone deacetylase (HDAC) inhibitors. (A–B) mRNA expression levels of dentin sialophosphoprotein (Dspp), Runt-related transcription factor 2 (*Runx2*) (A), and alkaline phosphatase (Alp) (B) were assessed in MDPC23 cells on day five after treatment with or without HDAC inhibitors (suberoylanilide hydroxamic acid [SAHA], trichostatin A [TSA], and SP1-PTD) at the indicated concentrations. (C) Mineralization capacity was evaluated using Alizarin red S staining on day 12 with the respective HDAC inhibitors in MDPC23. (D) Cell viability was evaluated in MDPC23 cells after 24 h of treatment with or without SP1-PTD at varying concentrations (#p=0.0313). (E) The cytotoxicity of SAHA (#p=0.0286),TSA (#p=0.0228), and SP1-PTD (#p=0.0695, ns) was evaluated in MDP23 cells after 24 h of incubation at the specified concentrations. Data are shown as mean ± standard deviation (n=3). *p<0.05 by Mann–Whitney U test, #p<0.05 by Kruskal–Wallis test.

To evaluate the therapeutic potential of SP1-PTD, its effects on odontoblast differentiation were compared to those of the classical pan-HDAC inhibitors, SAHA, and TSA (Figure 6). Odontoblast marker expression (*Dspp*, *Runx2*, and *Alp*) was quantified in MDPC23 cells treated with increasing concentrations of each HDAC inhibitor. SP1-PTD treatment resulted in a dose-dependent upregulation of all three genes, with maximal increases of 14-fold (p=0.05) for *Dspp*, 2.6-fold (p=0.05) for *Runx2*, and 1.8-fold (p=0.05) for *Alp* at 20 μM (Figures 6A and 6B). In contrast, SAHA elevated *Runx2* expression to levels comparable to SP1-PTD but concurrently suppressed *Dspp* expression (Figures 6A and 6B). TSA showed minimal or inhibitory effects on three genes under the tested conditions. These transcriptional trends were corroborated by mineralization assays: SP1-PTD treatment resulted in a more pronounced increase in mineralized nodule formation than SAHA or TSA, as evidenced by Alizarin red S staining (Figure 6C). Together, these findings indicate that SP1-PTD is a more effective inducer of odontoblast differentiation than nonspecific pan-HDAC inhibitors.

The cytotoxicity profile of SP1-PTD was compared with those of other HDAC inhibitors using MDPC23 cells. SP1-PTD treatment at concentrations ranging from 0.1 to 20 μM did not show cytotoxic effects; Instead, it promoted cell proliferation in a dose-dependent manner (Figure 6D). A reduction in cell viability was observed at 50 μM, although viability remained comparable to levels measured at lower concentrations (0.5, 1, and 5 μM) (Figure 6D). In contrast, SAHA and TSA induced a dose-dependent increase in the cytotoxicity across the tested dose range. These findings indicate that SP1-PTD has significantly lower cytotoxicity than other pan-HDAC inhibitors, such as SAHA and TSA (Figure 6E).

## Discussion

This study identifies SP1-PTD as a first-in-class peptide inhibitor that promotes odontoblast differentiation by selectively disrupting the HDAC4–SMRT interaction. In contrast to conventional pan-HDAC inhibitors, SP1-PTD achieves robust pro-differentiation effects while showing markedly reduced cytotoxicity. These findings highlight the therapeutic potential of this targeted approach, as SP1-PTD consistently enhanced odontoblast differentiation across multiple cellular models, from murine dental papilla cells to primary HDPCs.

The mechanisms of action of SP1-PTD represent a paradigm shift in HDAC inhibitor design. Unlike conventional inhibitors that target the conserved catalytic domain, SP1-PTD specifically disrupts the protein–protein interaction between HDAC4 and SMRT.^20^ This specificity is crucial given that HDAC4, a class IIa HDAC, lacks intrinsic deacetylase activity and instead functions as a scaffold for recruiting the HDAC3–SMRT complex.^11^ Mechanistic analyses conducted in this study demonstrated that SP1-PTD treatment enhanced the acetylation of both the RUNX2 protein and histones at the *Dspp* promoter. SP1-PTD simultaneously stabilized RUNX2 and increased chromatin accessibility at odontogenic promoters by preventing recruitment of the HDAC3–SMRT complex, providing a mechanistic explanation for the robust transcriptional activation observed.

The therapeutic benefits of SP1-PTD compared to pan-HDAC inhibitors are significant. While prior studies have shown that agents such as SAHA or TSA can induce partial odontogenic effects,^13,14^ our findings demonstrated that SP1-PTD consistently outperformed these inhibitors in both efficacy and safety. Rather than producing paradoxical or gene-specific effects, as reported for SAHA, SP1-PTD uniformly enhanced multiple odontogenic markers. Importantly, SP1-PTD showed a clear safety advantage, showing no cytotoxicity at effective concentrations, in contrast to the well-documented dose-dependent toxicity of pan-HDAC inhibitors. This favorable safety profile may be attributed to the selective disruption of protein–protein interactions, which preserves the broader catalytic functions of HDACs while specifically inhibiting HDAC4-mediated repression of odontogenic genes.

The clinical implications of these findings are also significant. Current pulp capping materials demonstrate limited regenerative capacity and frequently fail to achieve complete dentin bridge formation. Efforts to develop bioactive agents capable of inducing genuine regeneration have been impeded by safety concerns, particularly regarding pan-HDAC inhibitors, which are associated with systemic toxicities such as thrombocytopenia, cardiotoxicity, and anemia.^17-19^ SP1-PTD overcomes these limitations by providing a targeted approach that promotes robust odontoblast differentiation without incurring these toxic effects. Furthermore, the peptide-based nature of SP1-PTD is well-suited for localized delivery, enabling its integration into dental materials or its application as a pulp-capping agent.

Several limitations warrant consideration. First, all experiments were performed *in vitro*, and the efficacy of SP1-PTD in promoting dentin regeneration *in vivo* has yet to be established, and the complex microenvironment of the dental pulp, encompassing immune interactions, vascularization, and extracellular matrix dynamics, may modulate its activity. Second, although histone acetylation at the *Dspp* promoter was demonstrated, comprehensive genome-wide analyses of chromatin accessibility are necessary to elucidate the specificity of SP1-PTD. Third, timing of differentiation is likely context-dependent. Odontogenic markers such as *Runx2* and *Dspp* responded earlier in our cellular models than has been reported in some other studies, suggesting that stage- or species-specific factors may influence the observed kinetics. This earlier response is likely attributable to the specific characteristics of our cellular models and induction conditions, as previous studies have also demonstrated that the timing of odontogenic differentiation marker expression can vary depending on cell type, stage of differentiation, and external modulators.^21-23^ Therefore, the kinetics observed in our study reflect context-dependent, model-specific regulatory dynamics, rather than experimental artifact. Consequently, determining the optimal timing for SP1-PTD administration during odontoblast differentiation will be critical to maximizing its therapeutic efficacy. The use of both murine dental papilla-derived cells (MDPC23, mDP) and primary human dental pulp cells further underscores the robustness of these findings and enhances their translational relevance.

Future research should focus on several critical areas. *In vivo* validation employing dental injury models is essential to confirm the regenerative potential of SP1-PTD. The development of appropriate delivery systems, such as injectable hydrogels or integration into restorative materials, is necessary to facilitate clinical translation. Moreover, given that HDAC4 interacts with myocyte enhancer factor 2C (MEF2C)—a transcription factor involved in skeletal and cardiac muscle differentiation and an upstream regulator of RUNX2—to regulate chondrocyte hypertrophy during skeletal development,^24,25^ SP1-PTD may have broader applications in skeletal regenerative medicine. Since HDAC4 suppresses chondrocyte hypertrophy by inhibiting the transcriptional activities of MEF2C and RUNX2,^26,27^ SP1-PTD could potentially enhance endochondral bone formation, thereby warranting further investigation in orthopedic contexts.

## Conclusion

SP1-PTD represents a significant advancement in developing epigenetic modulators for regenerative medicine. This peptide inhibitor achieves targeted promotion of odontoblast differentiation while avoiding the toxicities that have hindered the clinical application of HDAC inhibitors by selectively disrupting the HDAC4–SMRT interaction. The specificity and favorable safety profile of SP1-PTD position it as a promising therapeutic candidate for dental pulp regeneration and potentially other regenerative contexts involving HDAC4-mediated regulation.

## References

[1] 1 - Karunakaran S, Praveen N, Selvandran KE, Leburu A, Madhuram K, Arunkumar AR. Effectiveness of mineral trioxide aggregate and its modifications in inducing dentin bridge formation during pulp capping: a systematic review. J Conserv Dent Endod. 2025;28(3):222-30. doi:10.4103/JCDE.JCDE_848_24 10.4103/JCDE.JCDE_848_24 PMC12007749 40256699

[2] 2 - Camilleri S, Mcdonald F. Runx2 and dental development. Eur J Oral Sci. 2006;114(5):361-73. doi:10.1111/j.1600-0722.2006.00399.x 10.1111/j.1600-0722.2006.00399.x 17026500

[3] 3 - Chen S, Rani S, Wu Y, Unterbrink A, Gu TT, Gluhak-Heinrich J, et al. Differential regulation of dentin sialophosphoprotein expression by RUNX2 during odontoblast cytodifferentiation. J Biol Chem. 2005;280(33):29717-29727. doi:10.1074/jbc.M502929200. 10.1074/jbc.M502929200 15980071

[4] 4 - Ritchie H. The functional significance of dentin sialoprotein-phosphophoryn and dentin sialoprotein. Int J Oral Sci. 2018;10(4):31-6. doi:10.1038/s41368-018-0035-9 10.1038/s41368-018-0035-9 PMC6215839 30393383

[5] 5 - Komori T. Regulation of osteoblast and odontoblast differentiation by RUNX2. Oral Biosci. 2010;52(1):22-5. doi:10.1016/S1349-0079(10)80004-0

[6] 6 - Li S, Kong H, Yao N, Yu Q, Wang P, Lin Y, et al. The role of runt-related transcription factor 2 ( *Runx2* ) in the late stage of odontoblast differentiation and dentin formation. Biochem Biophys Res Commun. 2011;410(3):698-704. doi:10.1016/j.bbrc.2011.06.065 10.1016/j.bbrc.2011.06.065 21703228

[7] 7 - Kim HJ, Kim WJ, Ryoo HM. Post-translational regulations of transcriptional activity of RUNX2. Mol Cells. 2020;43(2):160-7. doi:10.14348/molcells.2019.0247 10.14348/molcells.2019.0247 PMC7057842 31878768

[8] 8 - Peserico A, Simone C. Physical and functional HAT/HDAC interplay regulates protein acetylation balance. J Biomed Biotechnol. 2011;2011:371832. doi:10.1155/2011/371832 10.1155/2011/371832 PMC2997516 21151613

[9] 9 - Nakatani T, Chen T, Johnson J, Westendorf JJ, Partridge NC. The deletion of Hdac4 in mouse osteoblasts influences both catabolic and anabolic effects in bone. J Bone Miner Res. 2018;33(9):1362-75. doi:10.1002/jbmr.3422 10.1002/jbmr.3422 PMC6457245 29544022

[10] 10 - Wang Z, Qin G, Zhao TC. HDAC4: mechanism of regulation and biological functions. Epigenomics. 2014;6(1):139-50. doi:10.2217/epi.13.73 10.2217/epi.13.73 PMC4380265 24579951

[11] 11 - Gallinari P, Di Marco S, Jones P, Pallaoro M, Steinkühler S. HDACs, histone deacetylation and gene transcription: from molecular biology to cancer therapeutics. Cell Res. 2007;17(3):195-211. doi:10.1038/sj.cr.7310149 10.1038/sj.cr.7310149 17325692

[12] 12 - Kwon A, Park HJ, Baek K, Lee HL, Park JC, Woo KM, et al. Suberoylanilide hydroxamic acid enhances odontoblast differentiation. J Dent Res. 2012;91(5):506-12. doi:10.1177/0022034512443367 10.1177/0022034512443367 22447851

[13] 13 - Jin H, Park JY, Choi H, Choung PH. HDAC inhibitor trichostatin A promotes proliferation and odontoblast differentiation of human dental pulp stem cells. Tissue Eng Part A. 2013;19(5-6):613-24. doi:10.1089/ten.TEA.2012.0163 10.1089/ten.TEA.2012.0163 23013422

[14] 14 - Duncan HF, Smith AJ, Fleming GJ, Partridge NC, Shimizu E, Moran GP, et al. The histone-deacetylase-inhibitor suberoylanilide hydroxamic acid promotes dental pulp repair mechanisms through modulation of matrix metalloproteinase-13 activity. J Cell Physiol. 2016;231(4):798-816. doi:10.1002/jcp.25128 10.1002/jcp.25128 PMC6410376 26264761

[15] 15 - Liu Z, Chen T, Han Q, Chen M, You J, Fang F, et al. HDAC inhibitor LMK-235 promotes the odontoblast differentiation of dental pulp cells. Mol Med Rep. 2018;17(1):1445-52. doi:10.3892/mmr.2017.8055 10.3892/mmr.2017.8055 PMC5780081 29138868

[16] 16 - Finnin MS, Donigian JR, Cohen A, Richon VM, Rifkind RA, Marks PA, et al. Structures of a histone deacetylase homologue bound to the TSA and SAHA inhibitors. Nature. 1999;401(6749):188-93. doi:10.1038/43710 10.1038/43710 10490031

[17] 17 - Subramanian S, Bates SE, Wright JJ, Espinoza-Delgado I, Piekarz RL. Clinical toxicities of histone deacetylase inhibitors. Pharmaceuticals. 2010;3(9):2751-67. doi:10.3390/ph3092751 10.3390/ph3092751 PMC4034096 27713375

[18] 18 - Ali A, Bluteau O, Messaoudi K, Palazzo A, Boukour S, Lordier L, et al. Thrombocytopenia induced by the histone deacetylase inhibitor abexinostat involves p53-dependent and -independent mechanisms. Cell Death Dis. 2013;4(7):e738. doi:10.1038/cddis.2013.260 10.1038/cddis.2013.260 PMC3730430 23887629

[19] 19 - Li W, Fu Y, Wang W. A real-world pharmacovigilance study investigating the toxicities of histone deacetylase inhibitors. Ann Hematol. 2024;103(8):3207-17. doi:10.1007/s00277-024-05691-2 10.1007/s00277-024-05691-2 38453702

[20] 20 - Park SY, Kim GS, Hwang HJ, Nam TH, Park HS, Song J, et al. Structural basis of the specific interaction of SMRT corepressor with histone deacetylase 4. Nucleic Acids Res. 2018;46(22):11776-88. doi:10.1093/nar/gky926 10.1093/nar/gky926 PMC6294515 30321390

[21] 21 - Sreenath TL, Cho A, MacDougall M, Kulkarni AB. Spatial and temporal activity of the dentin sialophosphoprotein gene promoter: differential regulation in odontoblasts and ameloblasts. Int J Dev Biol. 1999;43(6):509-16. 10610024

[22] 22 - Ching HS, Ponnuraj KT, Luddin N, Rahman IA, Nik Abdul Ghani NR. Early odontogenic differentiation of dental pulp stem cells treated with nanohydroxyapatite-silica-glass ionomer cement. Polymers (Basel). 2020;12(9):2125-39. doi: 10.3390/polym12092125 10.3390/polym12092125 PMC7569887 32957636

[23] 23 - Chen Y, Pethö A, Ganapathy A, George A. DPP promotes odontogenic differentiation of DPSCs through NF-?B signaling. Sci Rep. 2021;11(1):22076. doi: 10.1038/s41598-021-01359-3 10.1038/s41598-021-01359-3 PMC8586344 34764323

[24] 24 - Arnold MA, Kim Y, Czubryt MP, Phan D, McAnally J, Qi X, et al. MEF2C transcription factor controls chondrocyte hypertrophy and bone development. Dev Cell. 2007;12(3):377-89. doi:10.1016/j.devcel.2007.02.004. 10.1016/j.devcel.2007.02.004 17336904

[25] 25 - Dreher SI, Fischer J, Walker T, Diederichs S, Richter W. Significance of MEF2C and RUNX3 regulation for endochondral differentiation of human mesenchymal progenitor cells. Front Cell Dev Biol. 2020;8:81. doi:10.3389/fcell.2020.00081. 10.3389/fcell.2020.00081 PMC7064729 32195247

[26] 26 - Miska EA, Karlsson C, Langley E, Nielsen SJ, Pines J, Kouzarides T. HDAC4 deacetylase associates with and represses the MEF2 transcription factor. EMBO J. 1999;18(18):5099-107. doi:10.1093/emboj/18.18.5099 10.1093/emboj/18.18.5099 PMC1171580 10487761

[27] 27 - Vega RB, Matsuda K, Oh J, Barbosa AC, Yang X, Meadows E, et al. Histone deacetylase 4 controls chondrocyte hypertrophy during skeletogenesis. Cell. 2004;119(4):555-66. doi:10.1016/j.cell.2004.10.024 10.1016/j.cell.2004.10.024 15537544

